# Women’s experience of unplanned out-of-hospital birth in paramedic care

**DOI:** 10.1186/s12873-019-0267-9

**Published:** 2019-10-15

**Authors:** Belinda Flanagan, Bill Lord, Rachel Reed, Gail Crimmins

**Affiliations:** University of the Sunshine Coast, ML 40, Locked Bag 4, Maroochydore DC, QLD 4558 Australia

**Keywords:** Birth before arrival, Narrative, women’s perspective, paramedic, consent

## Abstract

**Background:**

Healthcare literature describes predisposing factors, clinical risk, maternal and neonatal clinical outcomes of unplanned out-of-hospital birth; however, there is little quality research available that explores the experiences of mothers who birth prior to arrival at hospital.

**Methods:**

This study utilised a narrative inquiry methodology to explore the experiences of women who birth in paramedic care.

**Results:**

The inquiry was underscored by 22 narrative interviews of women who birthed in paramedic care in Queensland, Australia between 2011 and 2016. This data identified factors that contributed to the planned hospital birth occurring in the out-of-hospital setting. Women in this study began their story by discussing previous birth experience and their knowledge, expectations and personal beliefs concerning the birth process. Specific to the actual birth event, women reported feeling empowered, confident and exhilarated. However, some participants also identified concerns with paramedic practice; lack of privacy, poor interpersonal skills, and a lack of consent for certain procedures.

**Conclusions:**

This study identified several factors and a subset of factors that contributed to their experiences of the planned hospital birth occurring in the out-of-hospital setting. Women described opportunities for improvement in the care provided by paramedics, specifically some deficiencies in technical and interpersonal skills.

## Background

Most births in Australia occur in hospitals, however, most recent statistics report that in 2016, 1237 births occurred prior to arrival at hospital; these account for 0.4% of all Australian births [1]. Comparatively, in Queensland 501 births occurred prior to arrival at hospital in 2016; accounting for 0.8% of all Queensland births [2]. Studies concerning women who birth before arrival at hospital report that between 28.2–91.5% of these births occur with paramedic attendance [3–5]. However, recent reports indicate a significant rise in unplanned out-of-hospital birth, and as a consequence, a rise in paramedic response to such cases [6, 7].

Births that occur in unplanned environments present with different risk factors and the ability of healthcare providers to identify women at risk is difficult to mitigate [6, 8]. Reports indicate that in developed countries, unplanned out-of-hospital births are predominantly attributed to a lack of antenatal care, socioeconomic disadvantage, geographical isolation, a multiparous mother, or a mother experiencing a precipitate birth [6, 9–11]. Unplanned out-of-hospital births are also linked to higher than usual rates of perinatal morbidity and mortality, and studies indicate that the complications experienced are largely due to preventable causes [3, 4, 9, 11, 12].

Notwithstanding the datum, there is little research that explores the experience of women who have an unplanned out-of-hospital birth. Yet, it is well known that in other birth settings the experience of birth has both short and long-term implications for a woman’s physical health and psychological wellbeing.

A woman’s expectation and experience of physiological birth are multidimensional. There are associations between the individual woman’s expectation and experience of birth that rely on the level of control and support provided. There are also associations between a woman’s self-efficacy and a positive or negative birth experience. A positive experience augments a woman’s sense of accomplishment, self-esteem and feelings of competence [13]. However, a negative childbirth experience can influence women’s emotional well-being including post-traumatic stress disorders and depressive moods [14]. It is also understood that the quality of the relationship with care providers, the attitudes of care providers, and the environment of care are the most relevant factors influencing women’s perception of their birthing experience [15, 16].

This study explored the dimensions of the birth experience within the context of the unplanned out-of-hospital environment. The contribution of women’s dialogue regarding the ‘unplanned out-of-hospital birth’ experience deepens the overall body of knowledge for care provided by paramedics in the out-of-hospital setting.

## Methods

Ethics approval was provided by the University of the Sunshine Coast Human Research Ethics Committee (HREC: S/15/825). The selection of recruited participants was purposeful and allowed for only those who had experienced unplanned out-of-hospital birth in paramedic care to be recruited. Participants requirements were:

- English speaking;
- over the age of 18 years at the time of the interview;
- birthed in the State of Queensland, Australia within the previous 5 years; and
- had an unplanned out-of-hospital birth in paramedic care.

Recruitment was achieved by advertising for participants utilising social media, on-line and print media/newspapers.

The narrative interview was conducted either in the participants home, via video link or phone and recorded electronically for post interview verification and accuracy. To achieve confirmability or auditability [17], which refers to the efficacy of the information reported, field notes, transcripts, audio recordings, email correspondence, data reconstructions and data synthesis were included in an audit trail.

All women participating in this study gave a verbal account of their birthing experience. Narratives of birth stories provided rich, descriptive data and allowed the researchers the opportunity to represent the experience holistically [18, 19]. In the context of this research, birth stories were a reliable and credible form of datum. The spoken story presented a faithful description of how the women recalled their birth experience emotionally and cognitively. To ensure validity, the woman’s own words were crucial in interpreting the study findings. Placing the woman at the centre of understanding and knowledge development privileges a feminist approach [20] and allows women’s narratives to contribute to a paramedic body of knowledge.

Data was arranged according to the ‘diachronic organisation’ of narrativized events as outlined by Polkinghorne [21]. This type of analysis considers a description of an autobiographical account of lived experience and includes reference to when and why actions were taken, and the intended results of those actions. Thematic analysis was undertaken using the six phases of qualitative analysis recommended by Braun and Clarke [22]. This includes ‘Familiarisation and Coding’ (Phases 1 and 2), ‘Theme Development’ (Phase 3), ‘Reviewing and Defining Themes’ (Phases 4 and 5) and ‘Producing the Report’ (Phase 6) [22]. The result of the datum analysis was a retrospective explanation of the event.

## Results

The women interviewed described several aspects to the birth experience, including their experiences of both midwifery and medical interactions. However, the scope of this article will outline the results specific to the paramedic interaction. Overall 22 women were interviewed, participants’ ages ranged from 20 to 42 years and they were geographically located throughout the state of Queensland, Australia. All women had received the minimum Queensland standard of recommended antenatal care; four women were primipara (first time mothers) and 18 were multigravida (having their second or third baby). As interviews progressed ongoing analysis of the data occurred to recognise emerging themes and identify when saturation had been reached. Recruitment was discontinued at 22 interviews as no further themes were identified.

The women’s narratives described both positive and negative interactions with paramedics; in these episodes of care issues arose concerning clinical skills and interpersonal communication (Table 1).

**Table 1:** Themes and sub themes arising from women’s narratives

Themes	Communication	Consent	Respect and Empathy	Confidence
Sub themes	Effective and ineffective communication	Powerlessness Betrayal of trust	Autonomy	Skills and knowledge
	Interpersonal skills	Clinical assessment and interventions	Responsive to care needs	

It became evident that a woman’s positive birth experience related to the paramedic’s interpersonal skills and empathy, in addition to their clinical competence. Women who described the paramedic to be disrespectful, lacking empathy or possessing poor interpersonal skills, communicated a negative birth experience. Conversely, women who described positive birth experiences described the paramedic as skilled, responsive and respectful of their care needs.

### Communication

Women in this research provided examples of positive experience concerning paramedics’ communication during their unplanned out-of-hospital birth:

*Respondent 20 stated:*

*‘So, what made him amazing? His voice, he just kept on talking to me, kept on talking me through everything and when I got in the ambulance, I was alone with them but not with anyone that I knew, and it was just his voice was very calm and he just kept on talking and reassuring me.’*

In contrast, ineffective communication which included paramedics not listening to the woman’s wishes, particularly when she said she was pushing, were also shared. For the women, ‘not listening’ was understood as not being believed or respected.

*Respondent 11 stated:*

*‘I had to sort of raise my voice, be angry before they would listen to me.’*

*Respondent 9 stated:*

*‘I tried to get across that I wasn’t going to make it and when she was coming … I just don’t think that he believed what I was saying.’*

Some women felt the paramedic kept themselves at a distance and would not emotionally engage with them; that the paramedics’ approach was too clinical and technical or; that paramedics were just following steps in a policy or protocol instead of providing professional yet empathetic care.

*Respondent 11 stated:*

*‘Just no communication … I felt he was there to do his job.’*

Instead of paramedics using attentive, interpersonal communication skills, some women felt they were patronised and scolded whilst others felt a sense of guilt as they felt the paramedic blamed them for getting themselves into such a position.

*Respondent 12 stated:*

*‘He starts shouting orders and then he sits back there for quite some time holding my legs open, wouldn’t let me move, just holding them open … I was just like shocked at his behaviour.’*

*Respondent 8 stated:*

*‘When they arrived at my home, “Was this intentional, that you know, you had this baby at home or had you planned to go to the hospital?” … like they seemed almost annoyed that it looked like it was intentional.’*

Some women in this study stated that they had an expectation for open communication and involvement in decision-making. However, these findings revealed that some women felt a lack of participation in the patient-paramedic relationship, a lack of mutual respect and the failure of some paramedic’s listening to the woman’s views. In other words, a failure to provide patient-centred care.

### Consent

During pregnancy, women are provided with information from their healthcare professionals regarding birth options, risk and benefits of pain relief and care of the newborn. This information is necessary for women to make informed decisions during pregnancy about their birth and postpartum care.

In this research, some women identified specific areas of concern in relation to the provision of informed consent. The word ‘consent’ was not specifically used; however, their descriptions denoted a lack of consent. Some women were aware that permission should have been sought from them prior to procedures being conducted or prior to physical assessments; this also extended to procedures being carried out on the baby without parental consent. In addition, some women reported a lack of information provided by paramedics for them to make informed decisions about their care. Some women reported that at times they implied consent when they believed adhering to paramedic procedures for managing birth was a requirement, as no choice or discussion of options were presented. Some women were distressed – even angry – when care options progressed without actual discussion or input as to what they preferred or had considered as part of their birth plan were not regarded or valued.

*Respondent 5 stated:*

*‘I got in the ambulance, contraction started, and she started strapping up my arm to put in an IV and I didn’t know what she was doing. And I looked at her and I’m like, “What are you doing?” She goes, “I’ve got to put this in?” I said, “Well, can you at least wait until my … contraction has stopped?”’*

A lack of consent - while assessing the woman for signs of imminent birth - was a theme that emerged from the women’s narratives. While paramedics are not authorised to perform any type of vaginal examination to assess progress, a visual assessment for signs of imminent birth is sometimes required. This can be undertaken with maternal consent; particularly if the woman is pushing and a decision to transport or stay on-scene and birth the baby safely is required.

Two respondents described a lack of privacy and feeling violated because of a lack of consent before this assessment took place.

*Respondent 11 stated:*

*‘It was sort of really uncomfortable and [the paramedic] was feeling down there … And I mean at the heat of the moment I didn’t really think anything of it because I was like in labour, but after giving birth and sort of thinking about what happened I sort of felt like I’d been to the vet, if that makes any sense … I felt yuck … I still feel violated because I think that he violated like my trust in an ambulance officer … I didn’t know how to react. I didn’t know what to do about it, but I just felt like someone was perving on me. That’s how I honestly felt for months.’*

Feeling violated was a term used by some women, this was in relation to the actions taken without consent and how the paramedic made them feel during the interaction. A trust relationship between women and healthcare professionals is reliant on the ability of healthcare professionals to communicate effectively. A trusting relationship between the woman and the paramedic involved the woman being seen and heard so that they could receive support and care on their own terms.

*Respondent 11 stated:*

*‘I felt like that my trust was abused because obviously, I thought the paramedic was doing what he was supposed to be doing but nobody knows what they’re supposed to be doing really … So, in your head it’s like... your brain is just confused as hell because it’s like this is an ambulance officer, I should be trusting this person.’*

Some women also expressed some concerns over a lack of consent in relation to procedures performed on the baby.

*Respondent 5 stated:*

*‘They took the baby’s blood sugar levels and didn’t tell me.’*

Some women seemed well-informed about practices such as delayed cord-clamping: this particular procedure is recommended in the relevant clinical practice guideline for paramedics in this setting [23]. However, several women reported that this was not adhered to regardless of the woman’s expressed wishes.

*Respondent 1 stated:*

*‘They never asked me … it was kind of clamp; do you want to cut it? … I don’t recall being asked.’*

Although in the minority, there were positive stories where women’s wishes were respected.

*Respondent 17 stated:*

*‘They were absolutely fantastic. We wanted delayed cord-clamping … when they said they were going to cut bub’s cord … I asked them not to and they were really accommodating.’*

Consent is a critical component of paramedic practice as it is within all fields of healthcare. However, the commentary provided by women in this research reveal that consent is infrequently sought when dealing with a maternity case.

### Respect and empathy

For paramedics, responding to sick and emotionally distressed patients creates a working environment characterised by high emotional load that may be conflicted by organisational priorities. For example, the need to minimise response and scene times. In maternity cases, woman-centred care is the practice of caring for women and their families in ways that are meaningful and valuable to the individual. This includes listening to, informing and involving women in their care.

This study identified that there is a recurring theme of a need for respect, recognition and acceptance of the importance of involvement in decision-making. The most widely cited element of respect, mentioned in some form by most women, was simply paying attention to their individual needs and valuing their opinion.

*Respondent 2 stated:*

*‘They were really kind and they listened to everything we wanted.’*

For the women, allowing for autonomy showed professional respect. These women explicitly indicated that respect involved recognising them as autonomous by allowing them to make or participate in their own decisions.

*Respondent 5 stated:*

*‘The biggest thing is mum knows what’s happening … Just listen to what the mother wants.’*

*‘I think my biggest thing is not – and this is clearly any medical professional – not feel like you’re being told what to do even if they think it’s in your best interest.’*

Women’s narratives also provided some examples of negative experience when women felt their embodied or intuitive knowledge was disregarded.

*Respondent 12 stated:*

*‘I said she’s coming, and he said, “No she’s not” … and that’s what he just kept saying to me every time I had a contraction; just breathe. And then that uncontrollable feeling of you know pushing... I could feel her crowning.’*

Some women in this research had both negative and positive experiences that involved having their needs heard and respected. When the experience was positive, some women felt confident that all considerations were made to prioritise them and their babies.

*Respondent 9 stated:*

*‘The guy that came in … who actually delivered her … I don’t know how to explain it but he’s just my hero, he was absolutely amazing. He came in and told me what was going to happen, what he was going to do, what stage I was at and so it wasn’t going to be long … it could have been incredibly scary … because it happened too quickly but also they gave us absolutely no need to [worry].’*

The experience of unplanned out-of-hospital birth was unexpected for all women interviewed in this study. The intention for all women was that they would birth in a hospital or birthing centre setting. This unexpected change of experience created anxiety for some, and some women stated they required reassurance and confidence in the care being provided. In a short space of time a relationship between the paramedic and woman was established, the women felt they needed this, they wanted the paramedic to do more than look after their physical needs, they wanted empathy.

*Respondent 11 stated:*

*‘I think a paramedic sort of just does, you know just does the steps, necessities to keep you alive and the baby alive. Like the other things are not important … if you were to sit there and … ask an ambulance officer like what is your main goal if you arrive at a lady in labour’s house, it would be to get her to hospital in one piece … it wouldn’t be to support her during her labour... emotion would not be involved in it.’*

The need for paramedics’ to be aware of and respect women’s birth choices while executing clinical skills in a way that didn’t violate mothers’ rights was an expressed need of many women. This premise is associated with the woman’s need to have control over her birth and the care which is provided.

*Respondent 20 stated:*

*‘So just being able to preserve those things that were special to me in my birth made me really happy and I wouldn’t have expected that they would have known things like that, but they were already doing it for me which was amazing.’*

### Confidence and trust in paramedic care

Women expressed mixed feelings about being confident in paramedics’ ability to manage their birth or birth complications, if they occurred. Some women expressed complete confidence in paramedics because the paramedic appeared confident in their own abilities and demeanour.

*Respondent 14 stated:*

*‘(Paramedics) made me feel comfortable and I wasn’t scared at all ever while I was birthing, I always felt very safe.’*

Yet others lacked confidence in the care provided by paramedics. Some women stated the paramedics not only appeared to be unsure of how to manage a birth, they explicitly told the women they were inexperienced and made attempts to transport them to hospital as soon as possible. This resulted in a negative interaction with paramedics and anxiety for the family.

*Respondent 3 stated:*

*‘One of the ambulance workers on an iPad or something … I don’t know, it looked like an App that might tell them or guide them... one had delivered two babies before and one had never delivered a baby before … I was thinking, you know, do they know what they’re doing.’*

Perceptions of education and training for paramedics relating to unplanned out-of-hospital birth was also a concern for some women. Some women not only expressed doubt in paramedics’ abilities, they also expressed concern for a perceived lack of training.

*Respondent 2 stated:*

*‘They had never delivered a baby before and they told us that and they knew what they were supposed to do by the textbook, the basics of it.’*

## Discussion

The relationship between the paramedic and the patient is complex, and other studies suggest that the paramedic-patient interaction should embrace the whole person without reducing the patient to be a ‘recipient of an objectified ambulance care’ [24]. Women, having received antenatal care throughout their pregnancy, have been encouraged by midwives to expect holistic care, be able to make informed decisions and to expect that attaining choice and control during the birth would be respected. This in some cases became an area of conflict when paramedics became the caregiver and entered the relationship with an expectation that the woman would surrender to the paramedic’s requests. Conversely, some women experienced this shift-of-power as positive; when the ambulance arrived, the women reported a sense of relief. They surrendered themselves and their situation to the hands of another and had trust in the paramedics’ competence when they were respected and felt involved in their care.

Some women also expected an emotional connection with the paramedic, not an emotionally distant technical interaction. The women’s narratives demonstrate that increased emotional competence and relationality positively impact the birthing experience. At that moment, when the birth is imminent, the woman appears as much concerned with a paramedics’ bedside manner as she is with their clinical skills. Holmberg [24] refers to this as ‘being important while involved’. *Being important* according to Holberg is evident when paramedics show the patient’s respect, and the patients are involved in what is happening.

Providing information to patients and obtaining informed consent is a skill paramedics’ must learn early in their training and apply consistently. There is an ethical and legal expectation that it is done and done well. The findings illustrate that some women were not afforded the basic right and dignities they deserved, and paramedics disregarded convention regarding the establishment of consent. Some women stated that their care needs and preferences were sometimes ignored or superseded with adherence to ‘ambulance policy’, which served to limit the women’s range of care options.

The scope of skills expected of paramedics has increased significantly over recent years, as has the scope of paramedics’ roles and responsibilities. There has been a historical focus within paramedic education on a biophysical approach to the provision of care [25]. However, when asked what patients value most from paramedic care, the reply identified the principles of ‘professionalism’ [25]. This includes confidence in care, clinical competence and a high level of clinical knowledge. Women also want person-centred care which is respectful and responsive to the preferences, needs and values of the individual patients themselves.

The reasons for the suboptimal interpersonal skills reported by the mothers could not be determined by this study. Given that paramedic involvement in childbirth is infrequent, the findings may reflect a default reliance on technical skills in an unfamiliar environment that is designed to mitigate risk to patient safety. In this “emergency management” mode of health delivery the paramedic may assume the primary decision-making role and perform interventions under the “doctrine of necessity” [26], which assumes that the patient cannot provide consent. Given the importance placed on the therapeutic benefit of interpersonal skills revealed by this study, further work is required to identify means of enabling paramedics to provide safe, effective and respectful care to birthing mothers.

This research illuminates the need for education and professional development of paramedics based on a model of care that is patient-centred, embodies respect and requires paramedics’ empathy and developed interpersonal communication skills.

## Conclusion

Paramedics attend cases that can be challenging, including those involving health emergencies and it may be inferred that there is a sense of urgency for a paramedic to manage a birth as they would when responding to any other urgent case. As evidenced in this research, some women do not consider childbirth to be a ‘health emergency’. While acknowledging that paramedics may have limited birth-management experience from a clinical point of view; women did have an expectation of care that included respectful communication and anticipated that they would be asked to provide consent for medical examination and procedures.

## Data Availability

The identifiable data that support the findings of this study are not publicly available. Restrictions apply to the availability of the non-identifiable data.
